# Editorial: New challenges and future perspectives in neurodegeneration

**DOI:** 10.3389/fnins.2022.1049721

**Published:** 2022-10-10

**Authors:** Zhenyu Li, Hamid R. Sohrabi, Tomas Sobrino, Daniel Romaus-Sanjurjo

**Affiliations:** ^1^Department of Pathology, Chongqing University Cancer Hospital, Chongqing, China; ^2^Murdoch University Centre for Healthy Ageing, Perth, WA, Australia; ^3^Health Research Institute of Santiago de Compostela (IDIS), Santiago de Compostela, Spain

**Keywords:** neurodegeneration, DYRK, white matter hyperintensity, amyotrophic lateral sclerosis, proteomics

Over the last decade, there has been growing interest and important developments in neurodegeneration research. These advances have been fostered by improvements in the research techniques that enable us to collect novel data understand the disease process(es) and underlying mechanisms better. In this Research Topic, we encouraged researchers to summarize their state-of-the-art methods, findings, and the main challenges that the field of neurodegeneration is facing.

The nucleus is a membrane-bound organelle within eukaryotic cells that houses the genome, regulates cellular activities, and plays critical roles in cellular homeostasis and disease (Guo and Fang, 2014). The characterization of the proteomic composition of nuclei could provide a wealth of information regarding disease processes which exist in biorepositories of non-fixed frozen human brain tissues across several neurodegenerative diseases.

Nelson et al. investigated the proteomic changes in nuclei in neurodegenerative diseases and highlighted the potential for brain cell type-specific nuclear proteomics to enhance the understanding of distinct cellular mechanisms that drove neurodegenerative disease pathogenesis.

Dual-specificity tyrosine phosphorylation-regulated kinases (DYRKs) are involved in multiple neural cellular functions, including DNA damage repair, cell survival, neuronal development, synaptic plasticity, etc. which play a potential role in neurodegenerative diseases (Lindberg and Meijer, 2021). In this topic, Santos-Durán and Barreiro-Iglesias pointed out that DYRK2 as a key player regulating cytoskeletal dynamics and axon growth placing this protein as a promising target for spinal cord and brain injury and regeneration.

White matter hyperintensity (WMH) is a highly prevalent MRI marker of cerebral small vessel disease (SVD) which predicts stroke and dementia risk and is used as a surrogate marker in clinical trials. However, whether reducing the progression of WMH is a valid therapeutic target and, if so, what kind of intervention to use and when to start, is still open to question, and should be the subject of future studies (Prins and Scheltens, 2015).

In this direction, Shi et al. provided the information to help researchers and clinicians quickly and comprehensively understand the hotspots and emerging trends within WMH studies as well as providing direction for future basic and clinical studies on WMHs.

Amyotrophic lateral sclerosis (ALS), also known as motor neuron disease, is characterized by the degeneration of both upper and lower motor neurons, which leads to muscle weakness and eventual paralysis (Hardiman et al., 2017). Several cases of coexisting ALS and Neuronal intranuclear inclusion disease (NIID) pathology have been previously described (Sugiyama et al., 2021), however, there have been no reports of only NIID pathology with an ALS phenotype. Fujita et al. described a case of NIID with an ALS phenotype and suggested that a small proportion of patients with NIID can manifest a clinical phenotype of ALS. Although skin biopsy is commonly used for the clinical diagnosis of NIID, it may also be useful to identify cases of NIID masquerading as ALS.

Overall, these series of articles within the present Research Topic have brought several interesting findings allowing the understanding of brain cell type-specific nuclear proteomics, DYRKs, WMH, and NIID with an ALS phenotype in a wide range of neurological conditions.

Brain cell type-specific nuclear proteomics includes DYRK1A triplication in the formation of the cerebral cortex begins at the onset of neurogenesis, which is relevant to the search for early therapeutic interventions in Down syndrome (Najas et al., 2015). Besides, RBM45 from nuclear proteomics, associates with a number of other RBPs primarily *via* RNA-dependent interactions in the nucleus, and suggest novel functions for this protein, and provide new insights into the contributions to neurodegeneration in ALS (Li et al., 2016). Taken together, Brain cell type-specific nuclear proteomics provide a potential role to enhance the understanding of distinct cellular mechanisms in neurodegeneration. Interestingly, patients with NIID can be identified by searching for abnormalities at the junction between gray and WMH on DWI in picture archive and communication system and subsequently confirmed by skin biopsy (Yu et al., 2019).

We expect that this topic expands our knowledge on the biological basis and clinical research as well as new challenges and future perspectives in neurodegeneration providing exciting insights into new therapeutic approaches to various neurological disorders.

## Author contributions

ZL and HS contributed to the drafting of the manuscript and reviewed the final manuscript. TS and DR-S contributed to the discussion. All authors approved the submitted version.

## Conflict of interest

The authors declare that the research was conducted in the absence of any commercial or financial relationships that could be construed as a potential conflict of interest.

## Publisher's note

All claims expressed in this article are solely those of the authors and do not necessarily represent those of their affiliated organizations, or those of the publisher, the editors and the reviewers. Any product that may be evaluated in this article, or claim that may be made by its manufacturer, is not guaranteed or endorsed by the publisher.
